# Current research status and future prospects of NLRP3 inflammasome in cardiovascular diseases: a bibliometric and visualization analysis

**DOI:** 10.3389/fcvm.2024.1407721

**Published:** 2024-07-03

**Authors:** Meiqi Miao, Yuanyuan Yang, Hailong Dai

**Affiliations:** ^1^ Department of Cardiology, The First Affiliated Hospital of Heilongjiang University of Traditional Chinese Medicine, Harbin, China; ^2^ Department of Acupuncture, Dongzhimen Hospital, Beijing University of Traditional Chinese Medicine, Beijing, China; ^3^ Department of Cardiology, Yan'an Affiliated Hospital of Kunming Medical University, Kunming, China

**Keywords:** cardiovascular diseases (CVDs), bibliometrics, CiteSpace, NLPR3 inflammasome, visualization analysis

## Abstract

**Background:**

Cardiovascular disease (CVD) is a leading cause of global mortality, with atherosclerosis (AS) contributing to its pathological basis. Inflammation plays a critical role in the pathophysiological process of AS, and the NOD-like receptor protein 3 (NLRP3) inflammasome has been extensively studied in this context. This study aimed to analyze the research status of the NLRP3 inflammasome in cardiovascular disease and provide research directions for further exploration in this field.

**Methods:**

Using the “Bibliometrix” and “CiteSpace” software, a total of 516 articles were retrieved from the Web of Science (WoS) database published between 2012 and 2023. The search query used the keywords “[“CVD” OR “cardiovascular disease”] AND [“NLRP3 inflammasome “OR “NLRP3”]”. Visual analysis was performed on authors, countries, institutions, journal sources, keywords, references, and future trends.

**Results:**

A total of 516 English articles were retrieved, showing an overall upward trend in annual publication volume with slight fluctuations. China, the United States, and Europe were the countries and regions with the highest number of published articles. Among them, China had the highest article count (170), while the United States had the highest citation count (18,664), centrality score (0.43), and h-index (90), indicating its influential role in this research area. These countries also possessed elite institutions, professional researchers, and high-impact journals, making them leading contributors in this field. The main pathogenic mechanisms of the NLRP3 inflammasome in CVD were identified as “oxidative stress”, “pyroptosis”, and “inflammation”. The most frequently studied signaling pathways included “NF-κB”, “IL-1”, and “C-reactive protein”. The most studied disease types were coronary heart disease, atherosclerosis, metabolic syndrome, and myocardial infarction. Additionally, research on the correlation between cholesterol markers and inflammatory indicators associated with NLRP3 inflammasome in CVD risk assessment has gained significant momentum, with the main mechanism being NLRP3/IL-6/hs-CRP and cholesterol lipoproteins emerging as a major keyword in this context.

**Conclusion:**

This study provides valuable insights into the research hotspots and emerging trends of the NLRP3 inflammasome in cardiovascular disease. The findings offer guidance for researchers and scholars in this field and facilitate the exploration of new research directions.

## 1 Introduction

Despite significant advancements in the diagnosis and treatment of cardiovascular disease (CVD), it remains a leading cause of global mortality. The latest statistics released by the American Heart Association (AHA) reveal that CVD is one of the primary causes of death worldwide (1). In 2019, there were a total of 9.6 million male and 8.9 million female deaths attributed to CVD, accounting for approximately 30% of global deaths, which may be attributed to China having the highest number of CVD-related deaths (2). Atherosclerosis (AS) is a chronic inflammatory disease of the vasculature. Mediated by various risk factors (3), it is the pathological basis of CVD and is characterized by a long course with mild early symptoms, often leading to missed diagnoses and delayed treatment. Multiple studies have confirmed that inflammation is one of the main factors in the pathophysiological process of AS. Pro-inflammatory states, associated with different inflammatory mediators, are closely related to endothelial dysfunction and the development of AS. Various cytokines and inflammatory factors interact in the vascular wall to respond to endothelial injury, abnormal lipid metabolism, and hemodynamic disorders, thereby inducing chronic inflammation in the vessel wall (4). Given that excessive inflammation, platelet activation, and endothelial dysfunction increase the risk of thrombosis in atherosclerotic cardiovascular disease (ASCVD) (5), anti-inflammatory therapy is therefore a feasible strategy and a new target for treatment of this patient population.

The NOD-like receptor protein 3 (NLRP3) inflammasome, a crucial component in the pathogenesis of AS, has been extensively studied in recent years as a major causative agent of cardiometabolic diseases (6). The NLRP3 inflammasome regulates the inflammatory response in the body and is composed of NLRP3, CARD, Caspase-1, and cysteine asparaginase recruitment structural domain (ASC). Activated NLRP3 can interact with CARD8 to activate Caspase-1, leading to pyroptosis (7). The landmark study of anti-inflammatory and antithrombotic effects in AS, the Canakinumab Anti-inflammatory Thrombosis Outcome Study (CANTOS), has opened a new era of anti-inflammatory treatment for ASCVD (8). Subsequent trials such as the Colchicine Cardiovascular Outcomes Trial (COLCOT) and the LoDoCo2 trial (9–12) have further confirmed the involvement of specific inflammatory pathways in human ASCVD and emphasized the NOD, LRR, and NLRP3 inflammasome-related pathways as effective therapeutic targets for alleviating ASCVD. Therefore, anti-inflammatory treatment targeting the NLRP3 inflammasome may become one of the most effective therapeutic approaches for cardiovascular disease. This study aimed to use visualization analysis software (“CiteSpace” and “Bibliometrix”) to explore the research trends and hotspots related to the NLRP3 inflammasome in cardiovascular disease over the past 12 years.

## 2 Materials and methods

“Bibliometrix” is a software program developed by Massimo Aria, Corrado Cuccurullo from the University of Naples, Italy, and Luigi Vanvitelli from the University of Campania. Written in R language, it features an intuitive and well-organized interface for conducting comprehensive bibliometric analysis. Functionalities include support for various database sources, performance analysis, and comparative analysis of visualization options (13). “CiteSpace” is a visualization software developed in 2003 by Professor Chaomei Chen of Drexel University. Programmed in Java, CiteSpace measures the literature in a specific field and constructs visual maps to represent it (14). Within CiteSpace software, each node represents an evaluated object. The larger the node diameter, the more entries it represents. Colors differentiate the years of publication. Lines connecting nodes reflect collaborative or co-citation relationships between entries, with the thickness of the lines indicating the degree of closeness in those relationships. Thicker lines signify stronger relationships (15). Centrality is a metric used to assess the importance of an element within the network. Elements with a centrality greater than 0.1 are depicted with a purple ring, highlighting their relative significance (16). When Q is greater than 0.3 and the mean profile value is greater than 0.5, it suggests a sufficiently significant clustering structure with good homogeneity, leading to convincing results (17). This software enhances understanding for relevant practitioners and provides crucial support for analyzing research hotspots and trends in a specific field. Therefore, in this study, we utilized both “Bibliometrix” and “CiteSpace” to visualize and analyze the literature pertaining to the NLRP3 inflammasome in CVD research. Our focus encompassed publication trends, sources, core authors and teams, countries and institutions, keywords, and research trends. Overall, we sought to provide a scientific basis for the systematic promotion of anti-inflammatory therapy in CVD.

### 2.1 Data sources

This study used the Web of Science (WoS) database, a well-established data source that provides databases and citation data in the life sciences, social sciences, physical sciences, and health sciences (13). WoS is a large and recognized database that contains abstracts and references of high-quality and influential scientific papers (18–21). The time span of this search ranged from 2012 to 01-01 to 2023-12-31. The search terms used were as follows: TS = [“CVD” OR “cardiovascular disease”] AND TS = [“NLRP3 inflammasome “OR” NLRP3 “]. The main types of literature chosen for this study were articles and reviews, limited to the English language, and resulted in 525 documents.

### 2.2 Data processing

**Figure 1:**
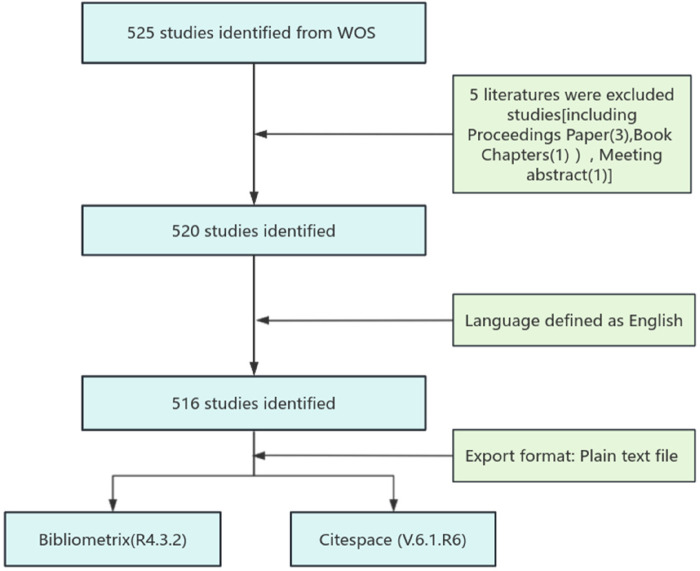
Detailed process for literature screening.

To ensure a comprehensive analysis, this study employed specific inclusion and exclusion criteria for articles retrieved from the WoS database. Included documents were restricted to the English language and full-length publications encompassing either articles or reviews. Conversely, materials such as proceeding papers, book chapters, and meeting abstracts were excluded. (See Figure 1 for the detailed flowchart).

The retrieved 516 documents from the WoS database were exported in plain text format (*.txt) and then imported into both the Bibliometrix (R4.3.2) software and CiteSpace (6.1. R6) for visualization and analysis.

## 3 Results

### 3.1 Descriptive statistics

Table 1 presents the key findings from the visual analysis, which will be discussed in more detail in the following sections.

**Table 1:** Main information.

Description	Results
Main information about data	
Time span	2012:2023
Sources (Journals, Books, etc.)	269
Documents	516
Annual growth rate %	39.68
Document average age	4.03
Average citations per doc	48.11
References	39,766
Document contents	
Keywords plus (ID)	1,801
Author's keywords (DE)	1,211
Authors	
Authors	3,004
Authors of single-authored docs	11
Authors collaboration	
Single-authored docs	12
Co-authors per Doc	6.78
International coauthorships %	25.58

### 3.2 Annual distribution of publications

**Figure 2:**
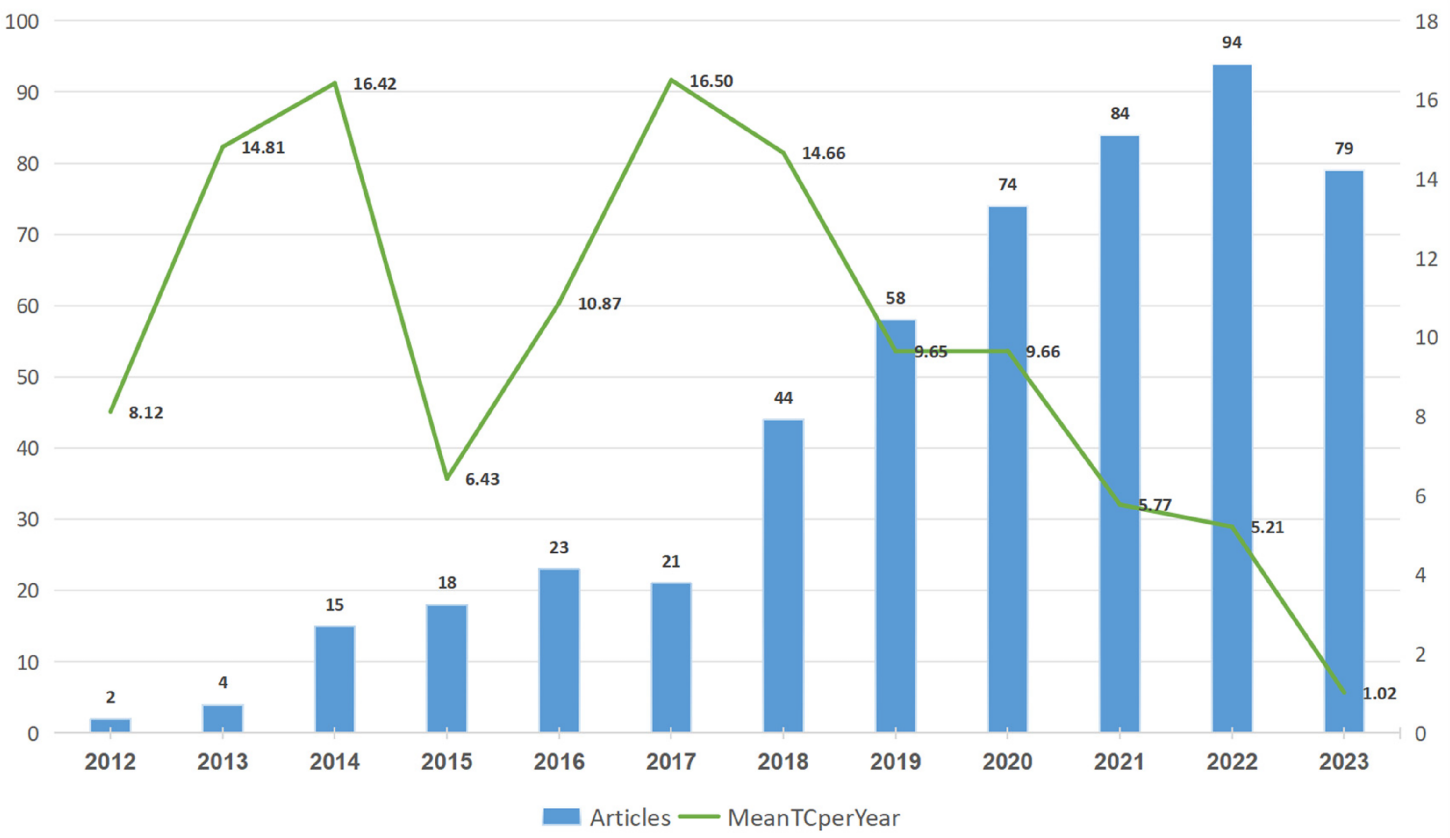
Annual distribution and average annual citations of publications from 2012 to 2023.

Figure 2 depicts the annual publication volume of CVD-related NLRP3 inflammasome research. The volume steadily increased from 2012 to 2016, followed by a transient decrease in 2017. A subsequent surge emerged from 2017, reaching a peak of 94 publications in 2022. The year 2023 saw a decline in publications. Overall, despite minor fluctuations, the publication trend for this field exhibited a clear upward trajectory. The average number of citations (mean TC) per year also demonstrated an upward trend from 2012 to 2014. However, it fluctuated between 2014 and 2016 before showing a downward trend from 2017 to 2023.

**Figure 3:**
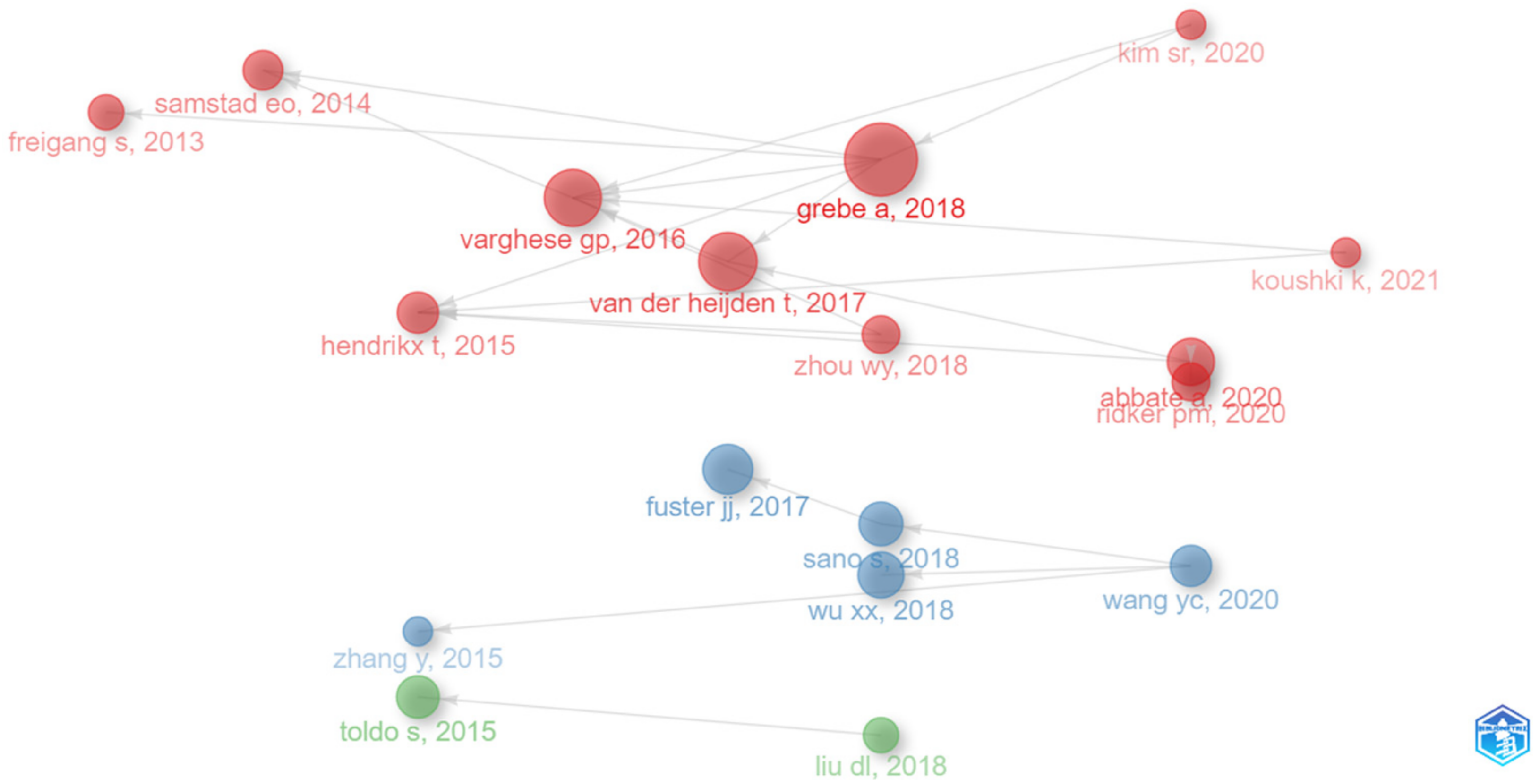
Historiograph.

To visualize the collaboration landscape of authors, we used “Bibliometrix” software and configured the following options: number of nodes: 20, local citation score (LCS) 10, global citation score (GCS) 50, and label: short ID (author, year). This resulted in a co-citation network (Figure 3) with 18 publications and 3 clusters, which may shed light on the observed fluctuation in publication numbers. Notably, in 2013, the FREIGANG team (GCS:243, LCS:11) initiated influential research on “CVD and inflammation” (Figure 2). Four years later, in 2017, publications by FUSTER (GCS:820, LCS:22) and VAN DER HEIJDEN (GCS:237, LCS:34) confirmed the pathogenic role of the NLRP3 inflammasome in AS and the potential of its inhibition for mitigating the disease. This likely contributed to the subsequent rise in research on “NLRP3 and CVD”.

### 3.3 Sources and cocited journals

This study analyzed publications on “CVD” and “NLRP3 inflammasome” from 2012 to 2023. Table 2 lists the top 10 journals based on the number of articles published, with the International Journal of Molecular Sciences leading with 26 articles (5.04%). Table 3 showcases the top 10 journals in terms of literature influence, encompassing a total of 269 journals included in the analysis. These influential journals included Frontiers in Cardiovascular Medicine (16 articles, 3.10%), Frontiers in Pharmacology (13 articles, 2.52%), Frontiers in Immunology (10 articles, 1.94%), Cells (9 articles, 1.74%), Circulation Research (9 articles, 1.74%), Frontiers in Physiology (9 articles 1.74%) Antioxidants & Redox Signaling (8 articles, 1.55%), Biomedicines (8 articles, 1.55%), and Cell Death & Disease (7 articles, 1.36%).

**Table 2:** Top 10 journals with publication volumes on “CVD” and “NLRP3 inflammasome” (2012–2023).

Sources	Articles	JCR	IF (2023)
International Journal of Molecular Sciences	26	Q1	5.6
Frontiers in Cardiovascular Medicine	16	Q2	3.6
Frontiers in Pharmacology	13	Q1	5.6
Frontiers in Immunology	10	Q1	7.3
Cells	9	Q2	6
Circulation Research	9	Q1	20.1
Frontiers in Physiology	9	Q1	4
Antioxidants & Redox Signaling	8	Q1	7
Biomedicines	8	Q2	4.7
Cell Death & Disease	7	Q1	9

**Table 3:** Top 10 influential journals from 2012 to 2023.

Sources	H-index	G-index	M-index
International Journal of Molecular Sciences	12	24	1.714
Circulation Research	8	9	0.8
Frontiers in Cardiovascular Medicine	8	13	1.143
Frontiers in Pharmacology	8	13	1.143
Antioxidants & Redox Signaling	7	8	0.7
Cell Death & Disease	7	7	0.875
Frontiers in Immunology	7	10	1
Frontiers in Cell and Developmental Biology	6	7	1.2
Oxidative Medicine and Cellular Longevity	6	7	0.667
Pharmacological Research	6	7	0.545

An analysis of the top 10 journals by publication volume (Table 2) revealed that CIRCULATION RESEARCH boasts the highest Impact Factor (IF) score of 20.1, signifying its exceptional influence within the field. Notably, 70% of these top-publishing journals were classified as Q1 journals, indicating their placement in the highest quartile based on citation impact. The remaining 30% fell under the Q2 category. In recent years, the H-index has emerged as a prominent metric for evaluating academic contributions and predicting future scientific output (22). Examining the top 10 journals based on literature influence (Table 3), we observed that the International Journal of Molecular Sciences held the highest H-index value (12). This underlined its significant impact within the field from 2012 to 2023.

**Figure 4:**
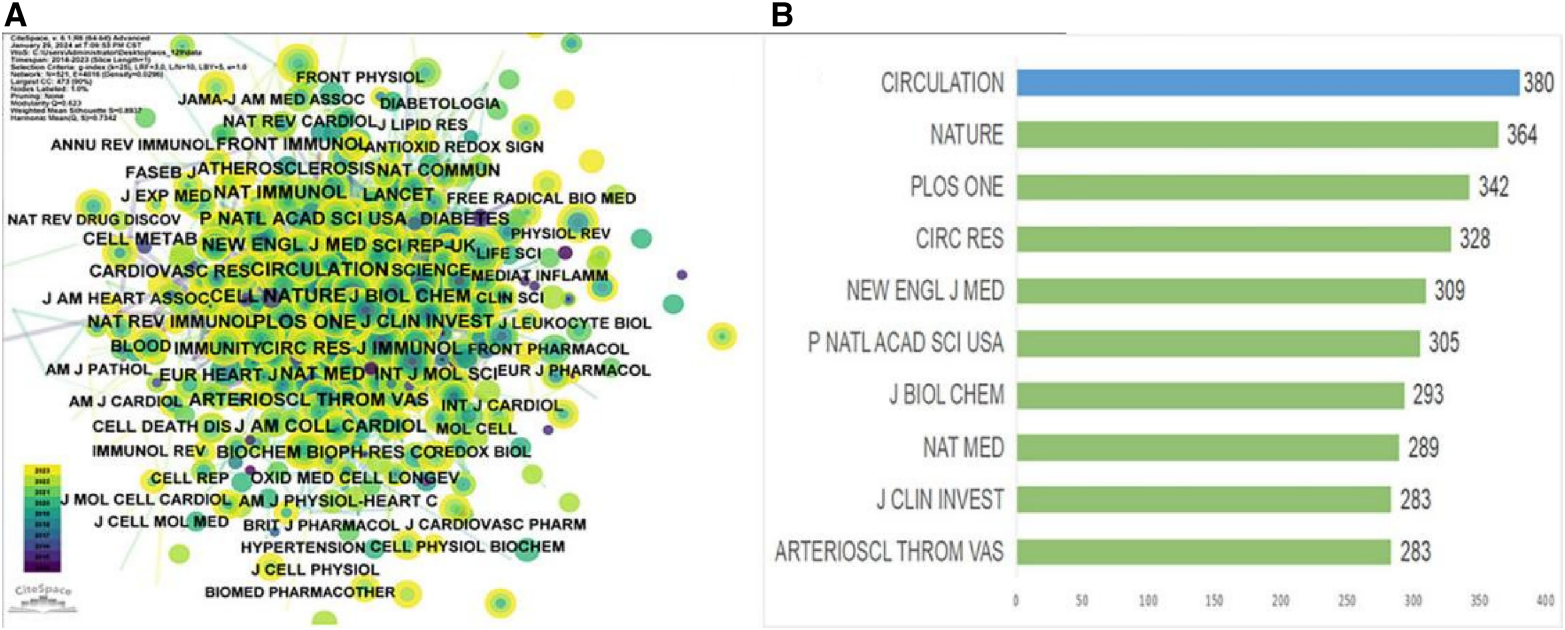
Visualization of journals and co-cited journals 2012–2023. (**A**) Co-citation journal co-publication cooperative network. (**B**) Top 10 journals in total citations from 2012 to 2023.

An analysis of journal co-citation network centrality revealed that CIRCULATION was the most frequently cited journal with a total of 380 citations, followed by NATURE with 364 citations, and PLOS ONE with 342 citations (Table 4, Figure 4B). Interestingly, among these top 10 most-cited journals (Table 4), the New England Journal of Medicine achieved the highest IF score of 158.5. Additionally, 80% were categorized as Q1 journals, indicating their placement in the highest quartile based on citation impact, with the remaining 20% falling under the Q2 category. The AMERICAN JOURNAL OF RESPIRATORY AND CRITICAL CARE MEDICINE (0.09) had the highest centrality score among the journals analyzed (Table 5). Free Radical Biology and Medicine (0.05) and Biochimica et Biophysica Acta—Molecular Cell Research (0.05) followed closely in centrality scores. These journals with high centrality scores demonstrated a significant influence within the field.

**Table 4:** TOP 10 Co-cited journals, 2012–2023.

Co-cited journals	Articles	JCR	IF (2023)
Circulation	380	Q1	37.8
Nature	364	Q1	64.8
Plos one	342	Q2	3.7
Circulation Research	328	Q1	20.1
New England Journal of Medicine	309	Q1	158.5
Proceedings of the National Academy of Sciences of the United States of America	305	Q1	11.1
Journal of Biological Chemistry	293	Q2	4.8
Nature Medicine	289	Q1	82.9
Arteriosclerosis, Thrombosis, and Vascular Biology	283	Q1	8.7
Journal of Clinical Investigation	283	Q1	15.9

**Table 5:** 2012–2023 Top 10 journals for centrality.

Sources	Centrality	JCR	IF (2023)
American Journal of Respiratory and Critical Care Medicine	0.09	Q1	5.6
Free Radical Biology and Medicine	0.05	Q2	3.6
Biochimica et Biophysica Acta—Molecular Cell Research	0.05	Q1	5.6
Oxidative Medicine and Cellular Longevity	0.04	Q1	7.3
American Journal of Physiology-Heart and Circulatory Physiology	0.04	Q2	6
American Journal of Cardiology	0.04	Q1	20.1
Journal of Lipid Research	0.04	Q1	4
Cell Death and Differentiation	0.04	Q1	7
Biochemical Pharmacology	0.04	Q2	4.7
Molecular and Cellular Biology	0.04	Q1	9

### 3.4 Authors and co-cited authors

**Figure 5:**
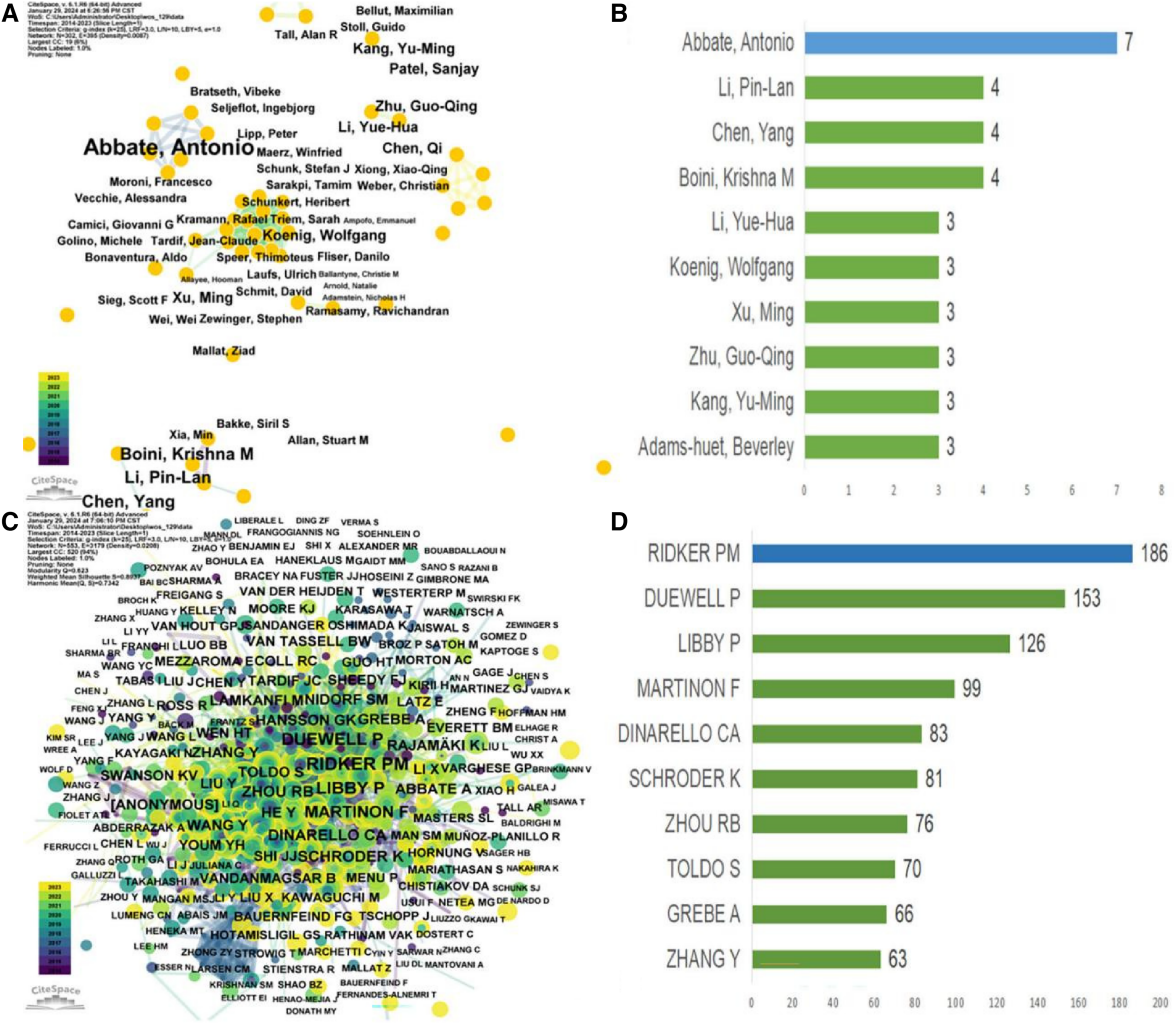
Analysis of author and co-cited author visualizations in relevant studies (2012–2023). (**A**) Co-authors; (**B**) The number of top 10 authors and their articles; (**C**) Co-authors co-occurrence; (**D**) The number of top 10 co-cited authors and their articles.

Table 6 presents the top 10 most prolific authors and their teams who contributed to publications on “CVD” and the “NLRP3 inflammasome” from 2012 to 2023. This analysis, encompassing 3,004 authors, revealed an average of 6.78 co-authors per paper and an international co-authorship rate of 25.58%. To further explore author collaboration patterns, a co-occurrence analysis was conducted using “CiteSpace” on authors with at least three publications (Figure 5). Abbate Antonio emerged as the most productive author, contributing to seven articles within the timeframe (Figures 5A,B). Following closely were Boini Krishna M, Chen Yang, and Li Pin-Lan, each with four publications. Centrality scores, displayed in Table 5, provide an additional indicator of authorial influence within the field. DINARELLO CA stood out with the highest score (0.11), exceeding the threshold of 0.1. This signified the significant impact of DINARELLO CA's research on the research landscape. Other prominent scholars included DUEWELL P (0.09), BAUERNFEIND FG (0.09), and MENU P (0.08).

**Table 6:** Most relevant authors.

Authors	Articles	Articles Fractionalized
Ridker PM	5	2.56
Roche HM	6	2.07
Zhang Y	14	1.84
Chen Y	8	1.54
Li X	7	1.38
Bornfeldt KE	3	1.33
Li PL	7	1.29
Latz E	5	1.29
Abbate A	7	1.20
Schertzer JD	3	1.20

Figure 5C provides a visual analysis of the co-cited author network, while Figure 5D showcases the top 10 most-cited authors based on their reference citations (Table 6). Notably, Ridker PM ranked among the most influential authors in the field of NLRP3 inflammasome research in the context of CVD, as evidenced by their high citation count. The CANTOS trial, led by Ridker PM's team, represents a significant breakthrough in anti-inflammatory treatment for atherosclerosis (8). This landmark study provided hitherto undocumented evidence that anti-inflammatory drugs targeting interleukin-1β (IL-1β) (canazumab) could effectively reduce the incidence of cardiovascular adverse events in patients with myocardial infarction, even when combined with lipid-lowering drugs. This finding ushered in a new era of anti-inflammatory therapy for ASCVD. Subsequent studies, such as COLCOT and LoDoCo2, have further confirmed the efficacy of colchicine in reducing cardiovascular risks among patients with chronic coronary heart disease and recent myocardial infarction who have received standard care (9–12). These studies have also highlighted the involvement of specific inflammatory pathways in ASCVD development. The NOD, LRR, and NLRP3 inflammasome-related pathways have been identified as promising therapeutic targets for achieving remission in ASCVD. In 2010, a groundbreaking discovery by Duewell et al. (23) employed novel observation techniques to reveal the presence of minuscule cholesterol crystals in the early stages of atherosclerotic plaque formation. These crystals were found to coincide with the infiltration of inflammatory cells and could activate the NLRP3 inflammasome, triggering Caspase1 activation, and inducing the release of abundant mature IL-1β. The authors hypothesized that cholesterol crystals function as endogenous molecules, activating the NLRP3 inflammasome and contributing to the progression of AS. Table 6 presents the top authors with the highest number of citations and their “Author Contribution Rate” to the respective articles. This metric reflects the proportional contribution of each author to the publications they are listed on.

### 3.5 Countries and institutions

**Figure 6:**
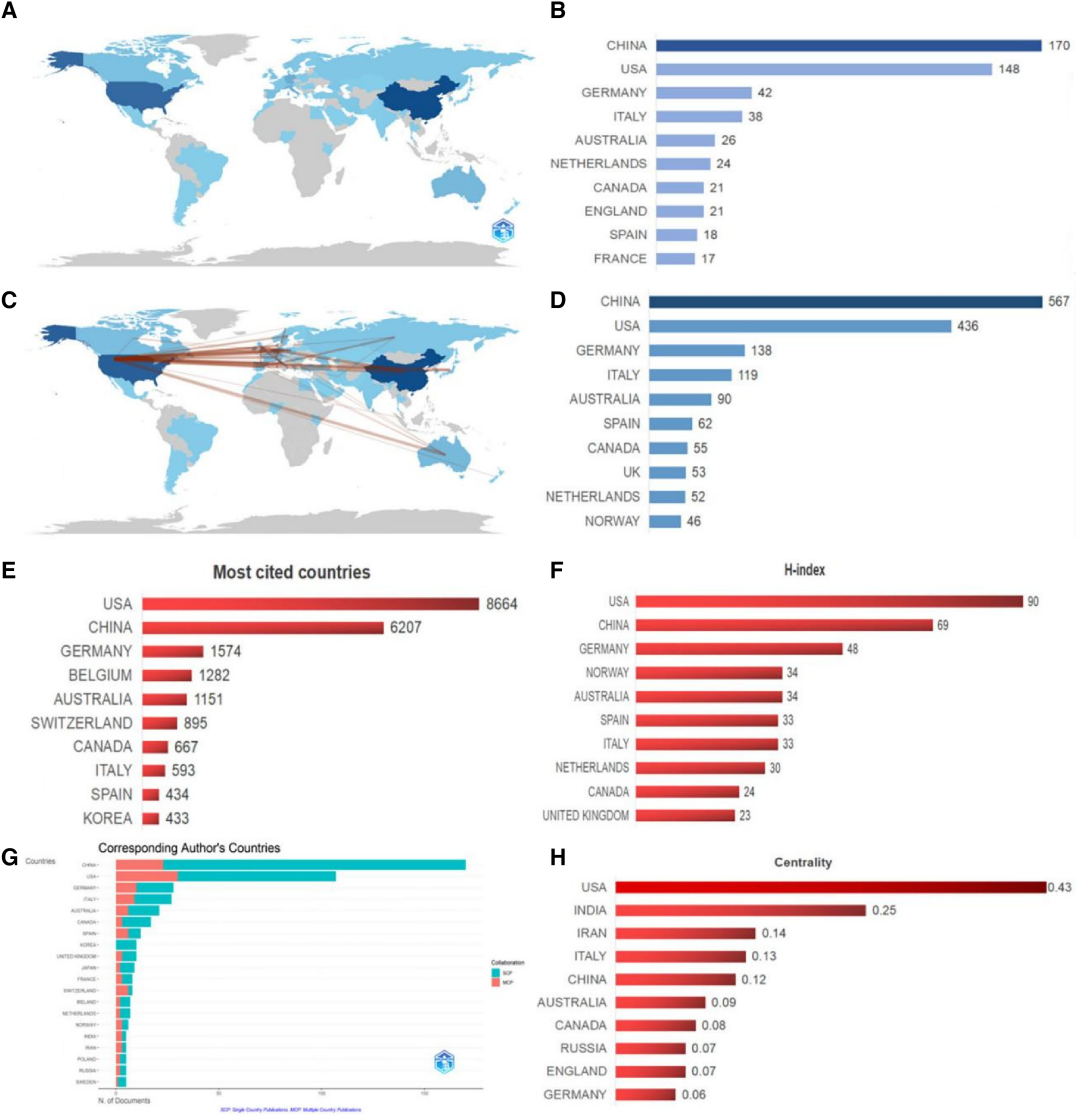
Visual analysis of countries involved in relevant studies from 2012 to 2023. (**A**) Number of publications per country. (**B**) The top 10 countries with the highest number of publications and the corresponding number of articles. (**C**) A map illustrating country cooperation, where lines represent collaborations between countries and the line thickness indicates the intensity of cooperation. (**D**) The top 10 countries with the highest level of cooperation. (**E**) Top 10 countries ranked by total citation frequency. (**F**) The top 10 countries in terms of the H-index. (**G**) A global map illustrating the intensity of cooperation among countries. (**H**) The top 10 countries with the highest centrality.

**Figure 7:**
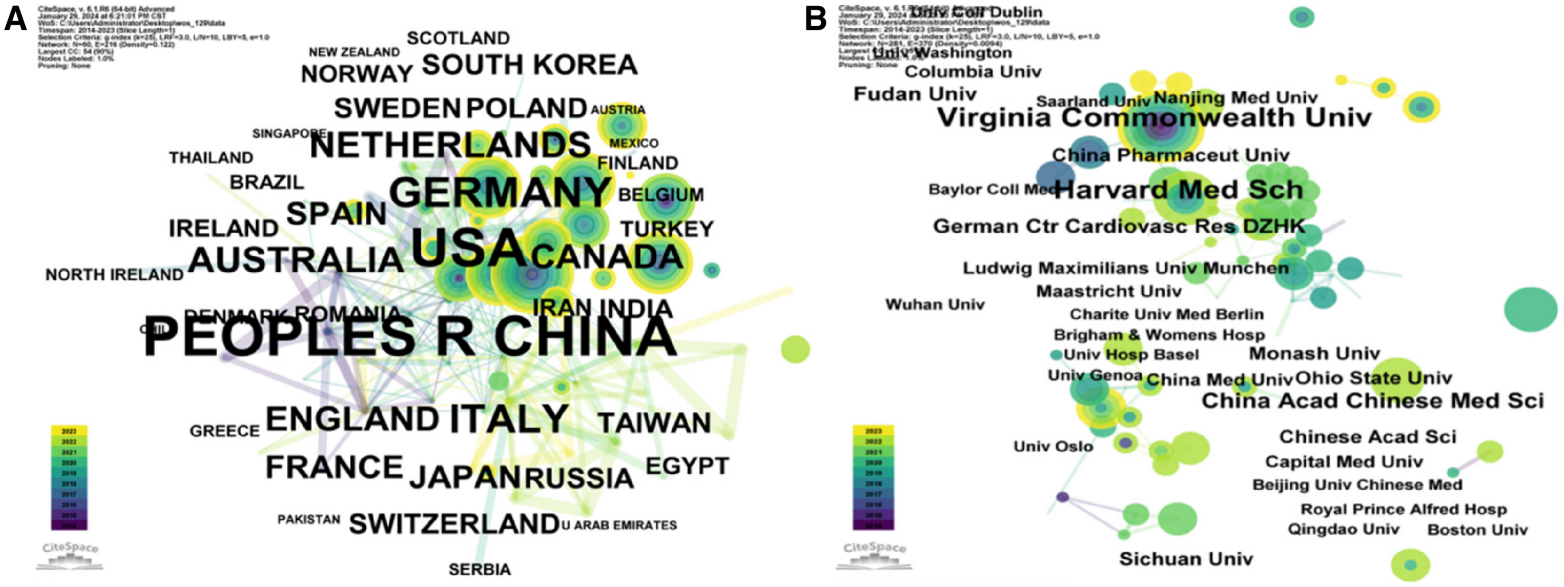
Country and agency map. (**A**) Co-occurrence map of countries. (**B**) Co-occurrence map of institutions.

Figures 6A,B and 7A reveal that research on the NLRP3 inflammasome in cardiovascular diseases has spanned 54 countries over the past 12 years. China led in publication volume with 170 articles (32.95%), followed by the United States (148/28.68%) and Germany (42/8.14%). An analysis of collaboration intensity (Figures 6C,D,G) indicated that China exhibited the strongest collaboration network, with a total collaboration strength of 567. The United States (436) and Germany (138) were next in terms of collaborative publications. The highest collaboration intensity was observed between the United States and China (19 publications), followed by the United States and Germany (16), and the United States and the Netherlands (12). Articles from the United States received the highest total citations (8,664) (Figure 6E), followed by China (6,207) and Germany (1,574). When considering centrality (a measure of a country's influence within the collaboration network), the United States (0.43) ranked highest, followed by India (0.25) and Iran (0.14) (Figure 6H). The H-index (an indicator of a country's research impact and productivity) was highest for the United States (90), followed by China (69) and Germany (48) (Figure 6F). Interestingly, although China had a slight edge in publication volume, the United States held a significantly higher H-index. By considering these publication, citation, and collaboration metrics, it is evident that China, the United States, and Europe have been the primary contributors to this field, with the United States holding a relatively prominent position.

**Figure 8:**
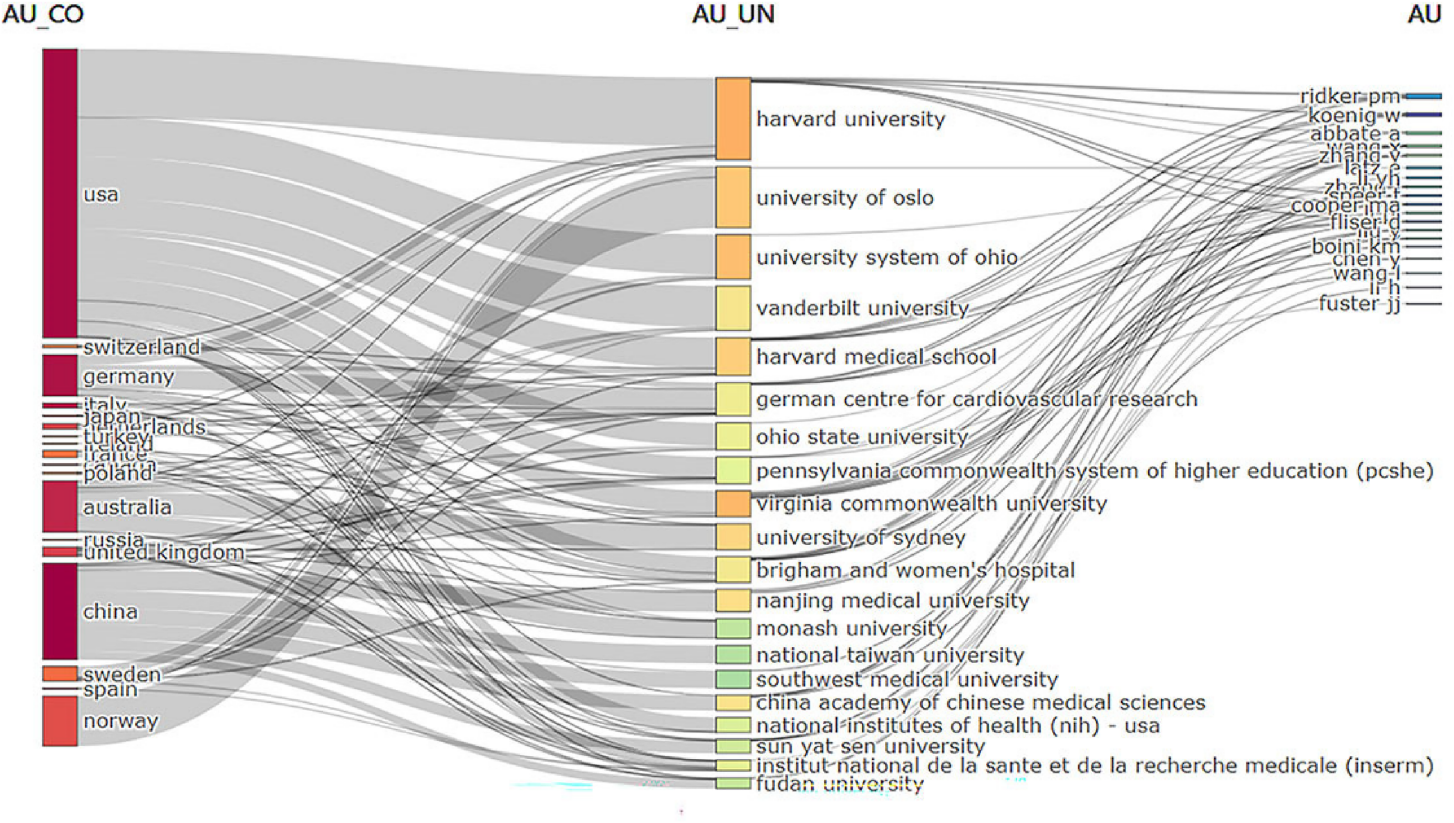
Three-region map analyzing national institutions and authors in relevant fields. Middle region: institution; Left region: countries; Right region: author.

An analysis of institutional contribution reveals that Harvard University led in publication output with 35 articles, followed by Harvard Medical School (25 articles) and the University of Oslo (24 articles) (Table 7). Centrality scores, which indicate an institution's influence within the collaboration network, were highest for the Chinese Academy of Sciences (0.34), followed by CIBER-Centro de Investigacion Biomedica en Red (0.16), and Harvard University (0.12) (Table 7). Figure 7B visually depicts the close collaborative ties among these institutions. A comprehensive network visualization is presented in Figure 8, encompassing authors, institutions, and countries involved in NLRP3 inflammasome research within the context of CVD.

**Table 7:** Ranking of the top 10 institutions conducting relevant research based on the number of studies and center status.

Rank	Affiliations	Counts	Rank	Affiliations	Centrality
1	Harvard University	35	1	Chinese Academy of Sciences	0.34
2	Harvard Medical School	25	2	CIBER—Centro de Investigacion Biomedica en Red	0.16
3	University of Oslo	24	3	Harvard University	0.12
4	University System of Ohio	24	4	Boston University	0.12
5	University of Sydney	17	5	Instituto de Salud Carlos III	0.12
6	Virginia Commonwealth University	17	6	German Centre for Cardiovascular Research	0.08
7	Brigham and Women's Hospital	16	7	Virginia Commonwealth University	0.07
8	Ohio State University	16	8	University of Queensland	0.06
9	German Centre for Cardiovascular Research	15	9	Egyptian Knowledge Bank (EKB)	0.06
10	Nanjing Medical University	14	10	Southern Medical University—China	0.06

### 3.6 Keywords

**Figure 9:**
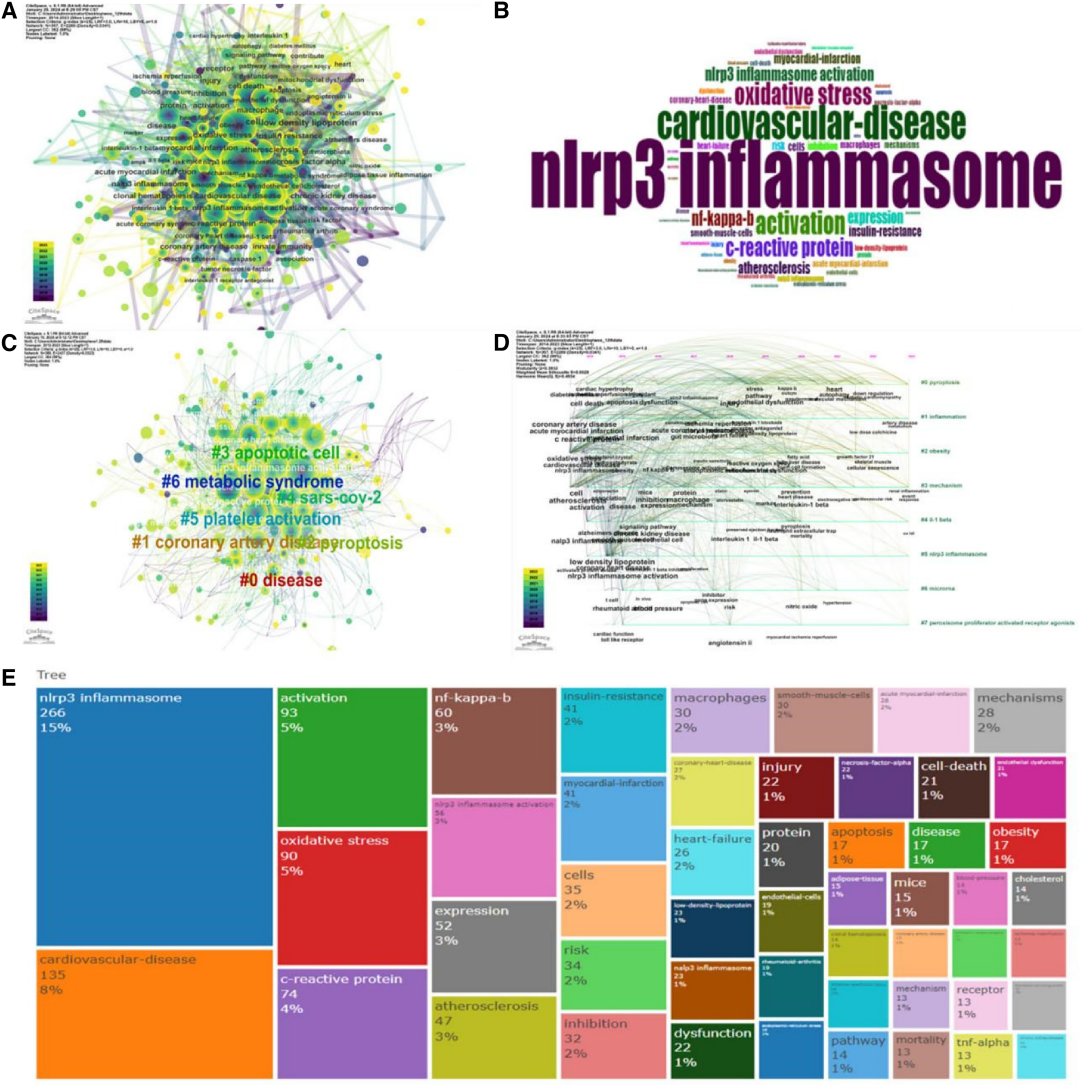
Visual analysis of keywords spanning the years 2012 to 2023. (**A**) Map illustrating the co-occurrence of keywords. (**B**) Word cloud depicting the keywords. (**C**) Diagram illustrating the co-aggregation of keywords. (**D**) Diagram displaying the timeline clusters of keywords. (**E**) Dendrogram depicting the relationships between keywords.

This study employed the “Bibliometrix” software to analyze keyword usage within the retrieved publications. Keywords appearing more than ten times were included in the analysis. A total of 1,211 keywords were extracted, with the top 50 most frequent keywords visualized in Figures 9B,E. Furthermore, co-occurrence analysis (Figure 9A) and cluster analysis (Figures 9C,D) were conducted on the keywords using “CiteSpace” software. This analysis resulted in the identification of nine distinct research clusters, each representing a specific research direction or field.

This analysis identified nine distinct research clusters based on keyword co-occurrence. Cluster 0, focusing on disease (*n* = 64 keywords), included terms like “acceptor” and “mechanism”. Cluster 1 comprised coronary heart disease (*n* = 56 keywords), encompassing keywords such as “IL-1”, “inflammation”, and “myocardial infarction”. Cluster 2 involved pyroptosis (*n* = 56 keywords), with terms like “apoptosis of cells” and “nrf2” appearing frequently. Cluster 3 focused on apoptotic cells (*n* = 41 keywords) with keywords including “reactive oxygen species” and “foam cell formation”. Cluster 4 comprised SARS-CoV-2 (*n* = 40 keywords), highlighting risk factors like “Sirt1” and diabetic complications. Cluster 5 examined platelet activation (*n* = 38 keywords), with terms like “homocysteine” and “vascular smooth muscle cells” present. Cluster 6 involved metabolic syndrome (*n* = 37 keywords), featuring keywords like “obesity” and “insulin resistance.” Cluster 7 was associated with chronic kidney disease (CKD) (*n* = 28 keywords), with “innate immunity” and “kanakulizumab” emerging as important keywords. Finally, Cluster 8 focused on endothelial cells (*n* = 24 keywords), with “signaling pathways” and “microRNA” being prominent terms.

Clusters 2, 3, 4, and 5 predominantly involved the cellular mechanisms and molecular pathways by which NLRP3 inflammasomes contribute to cardiovascular diseases. Keywords within these clusters highlighted terms like pyroptosis, apoptosis, and platelet activation. In contrast, clusters 0, 1, 6, and 7 primarily represented the major disease types investigated in the context of NLRP3 inflammasomes and cardiovascular disease, encompassing keywords such as coronary heart disease, metabolic syndrome, and chronic kidney disease. An analysis of keyword publication years revealed that obesity, ferroptosis, and inflammation were the most recently appearing keywords. This suggested that these topics represented current research hotspots in the field.

### References

3.7

**Figure 10:**
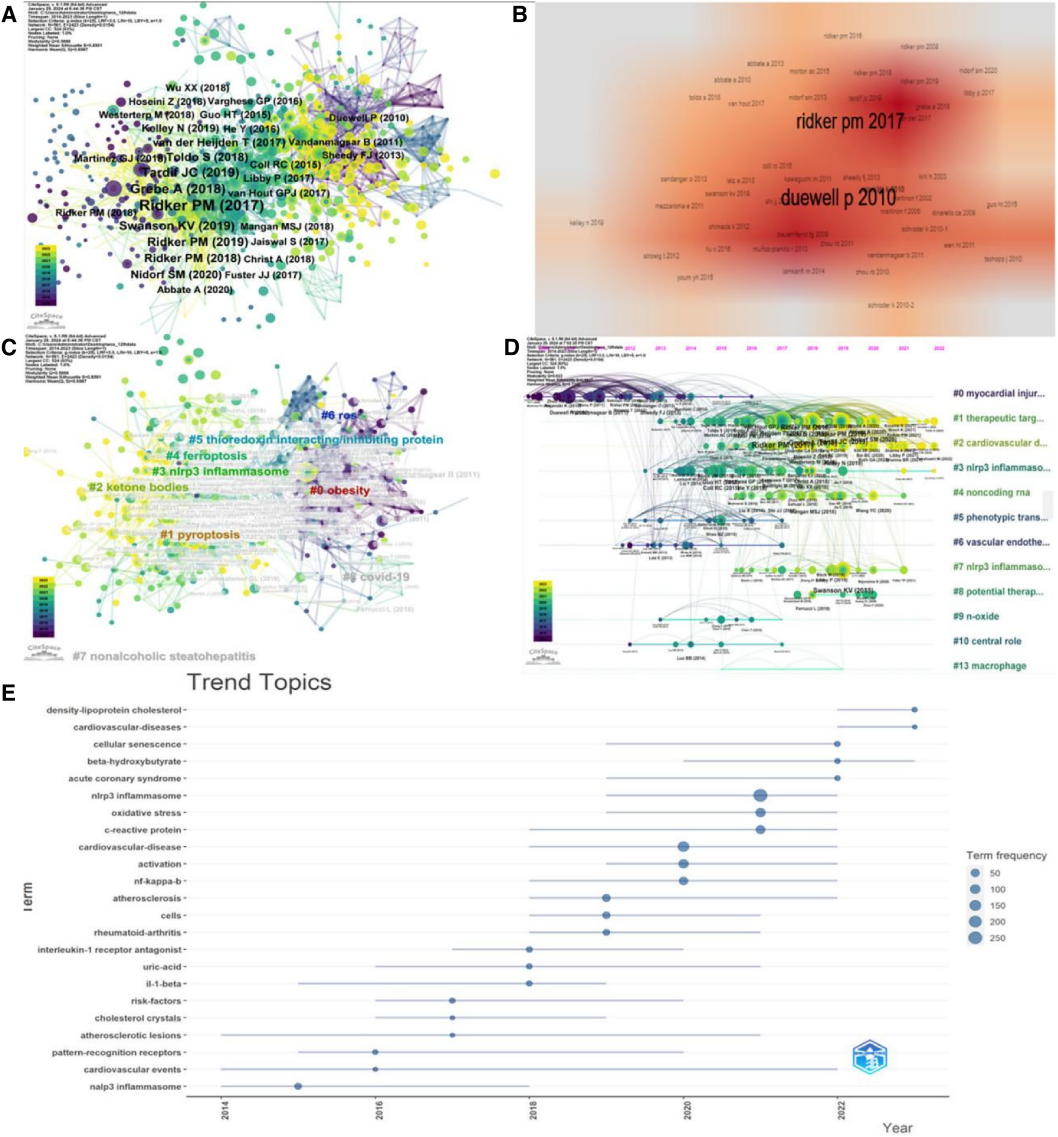
Visual analysis of cited references. (**A**) Co-cited literature network. (**B**) Density visualization of co-cited references. (**C**) Cluster analysis of co-cited references. (**D**) Timeline graph of cluster analysis. (**E**) Timeline distribution of topics.

**Figure 11:**
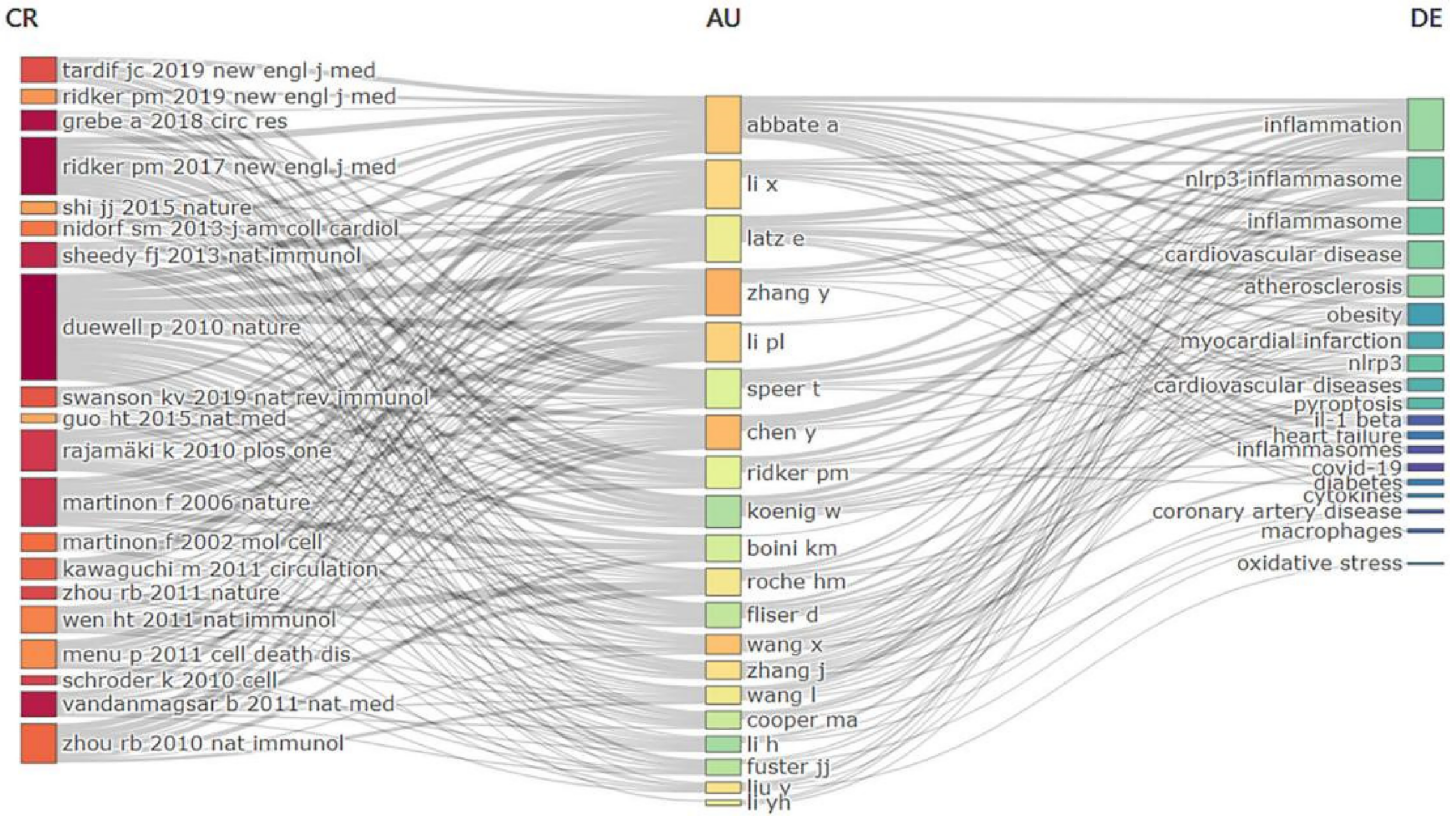
Three-field plot of references and keyword analysis in relevant fields. Middle region: author; Left region: references; Right region: keywords.

Citespace analysis revealed that four out of the top 10 most cited references (Figure 10, Table 8) focused on the association between NLRP3 inflammasome and atherosclerosis. Two others were large cohort studies investigating NLRP3 inflammasome-based anti-inflammatory therapy for cardiovascular diseases. Figure 11 illustrates the relationships among references, authors, and keywords in the field of CVD and NLRP3 inflammasome research. Notably, the most cited article by Duewell P (23). reported that endogenous cholesterol crystals activate the NLRP3 inflammasome, promoting the development of atherosclerosis. This finding suggests the potential benefit of combining lipid-lowering and anti-inflammatory therapies. It is now understood that activation of the NLRP3 inflammasome leads to Caspase-1 activation and subsequent secretion of inflammatory factors IL-1β and IL-18. Elevated levels of IL-1β have been linked to the severity of atherosclerotic disease (24). IL-1β stimulates various responses: synthesis and secretion of other cytokines and chemokines, activation of macrophages and lymphocytes, promotion of vascular smooth muscle cell migration and proliferation, enhanced cell-cell interactions, triggering of cell apoptosis, and contribution to extracellular cholesterol accumulation. Additionally, deposited crystals induce lysosomal disruption and trigger reactive oxygen species production in macrophages, further activating the NLRP3 inflammasome. Simultaneously, Caspase-1 activation can induce pyroptosis, a form of programmed cell death triggered by pathogens or endogenous factors, in monocytes and macrophages (25). This process leads to the release of tissue metalloproteinases by macrophages, compromising the stability of atherosclerotic plaques. These mechanisms create a positive feedback loop, enlarging the plaque area and reducing its stability. We then performed a cluster analysis of the citations based on the log-likelihood ratio (LLR), a statistical measure used to assess the association between variables. This analysis identified nine distinct clusters (Q = 0.5888, mean profile value = 0.6987) (Figure 10C). These clusters summarize two main aspects. Firstly, they highlighted the role of the NLRP3 inflammasome in the pathogenesis of cardiovascular disease. This includes the involvement of pyroptosis (#1), ketone bodies (#2), NLRP3 inflammasome (#3), ferroptosis (#4), and thioredoxin-interacting/inhibiting protein (#5). Secondly, the clusters represent the types of diseases studied in this field, such as obesity (#0), nonalcoholic steatohepatitis (#7), and covid-19 (#8). The timeline diagram (Figures 10D,E) revealed that recent research trends in this field focus on cardiovascular disease, targeted therapies, and lipoprotein cholesterol. Additionally, Figure 11 illustrates the interconnected network of authors, references, and keywords within the field.

**Table 8:** Top 10 co-cited references.

Authors	Journal	Year	Citations
Duewell P	Nature	2010	152
Ridker PM	New Engl J Med	2017	144
Grebe A	Circ Res	2018	64
Vandanmagsar B	Nat Med	2011	59
Martinon F	Nature	2006	56
Rajamäki K	PLoS One	2010	56
Schroder K	Cell	2010	56
Sheedy FJ	Nat Immunol	2013	56
Zhou RB	Nature	2011	50
Tardif JC	New Engl J Me	2019	47

### 3.8 Discipline distribution

**Figure 12:**
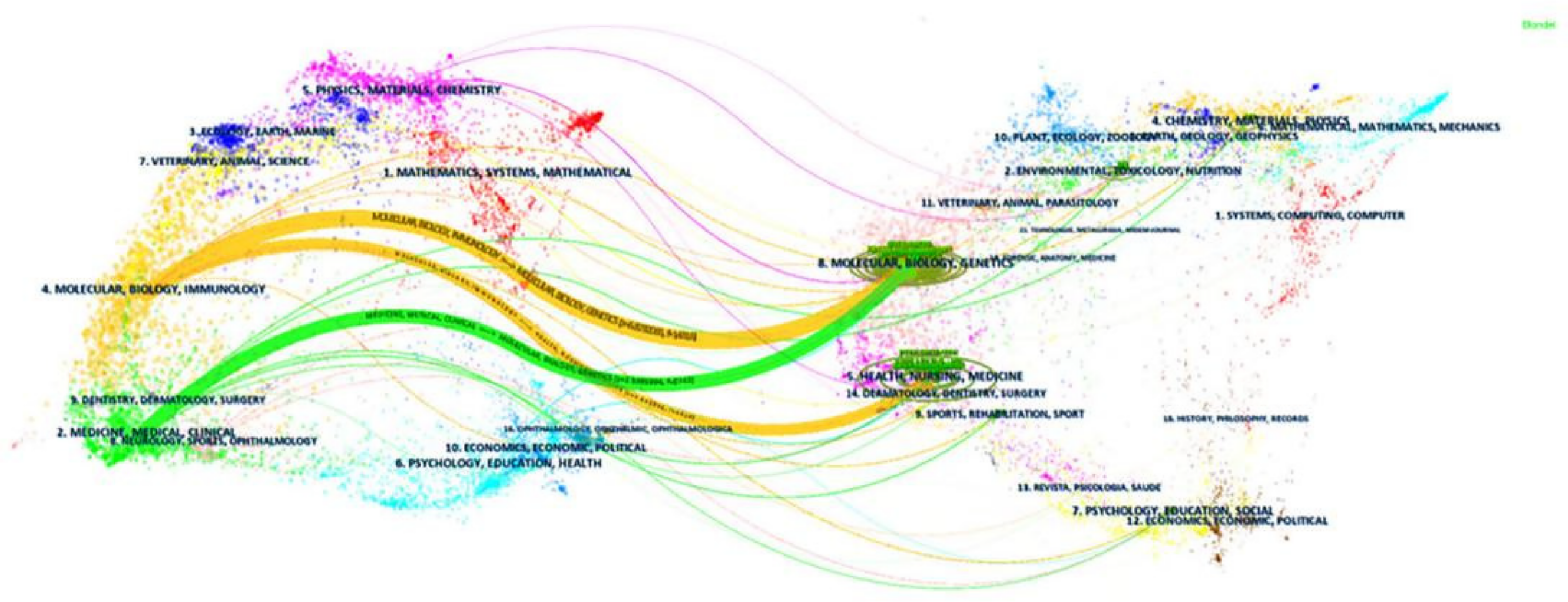
Visualization of the distribution of subjects. The left side represents the subject area covered by the citing journal, while the right side represents the subject area covered by the cited journal.

CiteSpace's double graph superposition function was employed to analyze the distribution of academic journals across disciplines (Figure 12). This analysis revealed two main citation paths within the network. Interestingly, the source articles themselves spanned a broad range of disciplines, including mathematics, medicine, clinical medicine, ecology, molecular biology, and immunology. Conversely, the cited references were primarily published in journals focused on biomedicine, such as biochemistry, biology, pharmacology, pharmaceutics, cardiology, immunology, general medicine, research methods, endocrinology, chemistry, and peripheral vascular disease.

## 4 Discussion

This study utilized Bibliometrix, CiteSpace, and Microsoft Excel to analyze 516 articles retrieved from the Web of Science Core Collection, focusing on NLRP3 inflammasome research in cardiovascular diseases. The investigation aimed to identify key research areas and emerging trends within this field. The analysis revealed a steady increase in publication volume over the past twelve years, with a particularly notable acceleration after 2017. This upward trajectory suggested that NLRP3 inflammasome research in cardiovascular diseases has reached a relatively mature stage of development.

Our analysis identified the United States, Europe, and China as the leading contributors to research in this field. While China boasted the highest publication output, the United States emerged as the frontrunner in terms of both citations and H-index. Notably, among the top 10 most prolific authors and institutions, 40% were affiliated with the United States, while the remaining 60% originated from China. This trend continued for institutions, with 90% of the top 10 being US-based, compared to just 10% from China. However, the landscape shifted when considering citations and centrality. Here, the US retained a strong presence with 60% of the top 10 authors, but its institutional dominance lessened, with 30% of the top institutions being US-based. These findings suggest that the United States harbors a robust network of globally recognized research institutions and accomplished scholars, contributing significantly to advancements in this field.

Analysis of co-cited literature and keywords offers valuable insights into the central themes and primary focus of current research. Additionally, examining frequently cited references helps establish the foundational knowledge and background of the field (26). Notably, this study observed a high degree of concordance between co-cited literature clusters and keyword clusters. This overlap suggests that the identified topics represent genuine research hotspots within the field.

Since 2017, research has increasingly focused on the role of NLRP3 inflammasome-mediated inflammatory responses in the development and progression of cardiovascular disease. Atherosclerosis serves as the underlying pathology for most CVD cases. Building upon the “inflammation theory” of AS proposed by Russell Ross in 1990, numerous studies have provided evidence that inflammation is a primary driver of AS and its complications (27). This process involves various cell types, including monocytes, macrophages, vascular endothelial cells, vascular smooth muscle cells, and T lymphocytes, as well as inflammatory cytokines such as C-reactive protein (CRP), interleukin-6 (IL-6), and IL-1β. These factors contribute to the activation of inflammatory signaling pathways throughout AS formation and development. At the molecular level, the formation of the NLRP3 inflammasome in macrophages plays a crucial role in propagating inflammation. The NLRP3/IL-1β/IL-6/high-sensitivity C-reactive protein (hs-CRP) classical inflammatory pathway is widely thought to be closely associated with an increased risk of vascular atherosclerosis (28). To validate the role of inflammation in CVD, researchers have conducted large-scale clinical trials targeting this mechanism. Examples include CANTOS, COLCOT, LoDoCo2, Cardiovascular Inflammation Reduction Trial (CIRT), and CLEAR Outcomes (8–12, 29–32).

Keyword analysis further highlighted the pivotal roles of NF-κB, oxidative stress, and CRP in NLRP3 inflammasome-related cardiovascular diseases. The NLRP3 inflammasome is a multi-protein complex requiring the coordinated regulation of two signaling pathways. First, a priming signal triggers the binding of stress molecules to Toll-like receptors on the cell membrane. This activates the NF-κB pathway, leading to increased expression of NLRP3 and pro-IL1β. Subsequently, upon recognition of a pathogen-associated molecular pattern (PAMP) or danger-associated molecular pattern (DAMP), a triggering signal induces the assembly of NLRP3, ASC, and pro-caspase-1 into functional NLRP3 inflammasomes. This results in the release of mature caspase-1, IL-1β, and IL-18. The NLRP3 inflammasome, a key driver of inflammation in atherosclerosis, can be activated through three main mechanisms. The first involves changes in potassium levels: either a decrease inside the cell, an increase outside, or the binding of ATP to a specific receptor. This disrupts the cell membrane, allowing NLRP3 agonists to enter and trigger inflammasome assembly. Secondly, instability or rupture of lysosomes can also activate the NLRP3 inflammasome. Finally, the production of reactive oxygen species, particularly from mitochondria, is another potential mechanism, although the exact details remain unclear (33). Macrophages exposed to low oxygen or cholesterol crystals experience further stress, leading to the formation of protein complexes. Activation of the NLRP3 inflammasome in these cells then triggers the release of IL-1β and IL-18. These cytokines activate various inflammatory cells and induce IL-6 production, which subsequently stimulates the liver to produce CRP. This process amplifies the inflammatory response within the artery wall (34). In heart failure, activation of the NLRP3 inflammatory mediates the release of pro-inflammatory mediators such as IL-1β and IL-18, considered significant contributors to myocardial fibrosis and cardiac dysfunction in heart failure (35). A study found activation of NLRP3 inflammasomes in a mouse model of myocardial ischemia/reperfusion injury and high levels of NLRP3 further aggravated myocardial injury (36). Toll-like receptors (TLRs) represent an important class of protein molecules involved in innate immunity. TLR4 has been demonstrated to facilitate NLRP3 inflammasome activation through the NF-κB pathway. Inhibition of TLR4 has been shown to suppress the inflammatory response, thereby attenuating myocardial ischemia-reperfusion injury (37). Several NLRP3 inflammatory vesicle inhibitors containing structural domains of the NACHT, LRR, and PYD protein family have been tested in animal models of acute myocardial infarction. Colchicine can act downstream of NLRP3 by inhibiting the polymerization of ASC-containing apoptotic speck-like proteins (38). In arrhythmias, the activation of NLRP3 inflammatory vesicles induces the upregulation of ultrarapid delayed rectifier K^+^ channels and shortening, which leads to myocardial potentiostatic inappetence and action potential duration. These two key factors contribute to cardiac electrical remodeling. It was found that increased pro-fibrotic signaling and fibrosis, as well as abnormal Ca^2+^ release from the sarcoplasmic reticulum, were strongly associated with arrhythmogenesis in wild-type mice fed a high-fat diet. In contrast, NLRP3 knockout in high-fat diet-fed mice (NLRP3 ^−/−^) prevented the upregulation of K^+^ channels and the evolution of electrical remodeling, the upregulation of pro-fibrotic genes, and the aberrant sarcoplasmic reticulum Ca^2+^ release induced by high-fat chow in wild-type mice. This suggests that the activation of NLRP3 inflammatory vesicles may be a key driver for the development of arrhythmias (39).

Furthermore, trend analysis identified lipoprotein cholesterol as a significant keyword with a strong association with NLRP3 inflammasomes in CVD research. The combined analysis of references and keywords suggested a thematic distinction within the field. Disease research appeared to be primarily focused on metabolic disorders, such as obesity and ASCVD. In contrast, mechanistic research seems to be concentrated on oxidative stress and pyroptosis pathways. It is well-established that inflammatory cytokines, mediated by NLRP3 inflammasomes, can exert both autocrine (acting on the same cell) and paracrine (acting on neighboring cells) effects on various cell types within metabolic tissues. This phenomenon is believed to contribute to the development of several metabolic disorders, including diabetes, obesity, atherosclerosis, cardiovascular disease, gout, and neurodegenerative diseases (40).

Since 2017, a new era of anti-inflammatory therapy for ASCVD has been ushered in by the groundbreaking CANTOS trial led by Professor Ridker. This study demonstrated that residual inflammation significantly increases the risk of recurrent events in ASCVD patients (8). Further support for targeting the NLRP3 inflammasome pathway as a therapeutic strategy for ASCVD comes from the COLCOT and LoDoCo2 studies published in 2021. Professor Ridker's most recent clinical trials (2023) (31, 32) demonstrated that combining anti-inflammatory treatment with statin-based lipid-lowering therapy could effectively reduce residual cardiovascular risk in CVD patients. These studies have established high-sensitivity C-reactive protein as a more reliable predictor of future cardiovascular events and mortality risk compared to low-density lipoprotein cholesterol (LDL-C). Collectively, these landmark clinical trials highlight the critical role of chronic systemic inflammation in ASCVD development and progression. Therefore, managing chronic systemic inflammation holds significant promise for reducing cardiovascular risk events.

Cholesterol has long been viewed as the primary culprit in ASCVD. However, research advancements now recognize ASCVD as a progressive inflammatory response, not just lipid buildup in arteries. In ASCVD patients, inflammation can be triggered by various factors beyond just metabolic conditions (diabetes) and autoimmune diseases. It can also originate from adipose tissue itself. In obesity, the accumulation of visceral fat leads to lower adiponectin levels, higher free fatty acids in the blood, and the release of inflammatory cytokines. This prolonged inflammation can damage the vascular endothelium (lining of blood vessels), alter blood flow dynamics, remodel the heart muscle, and ultimately contribute to atherosclerosis development and progression. These processes significantly impact heart structure and function (41). Studies have shown that a 10% weight loss can reduce hs-CRP levels by 40%, suggesting weight management offers cardiovascular benefits, likely due to improved inflammatory state (42). The SELECT trial, a groundbreaking global study, demonstrated that semaglutide 2.4 mg not only aids weight management but also reduces the risk of cardiovascular events (cardiovascular death, non-fatal myocardial infarction, and non-fatal stroke). This discovery represents a significant advancement in obesity treatment for patients with cardiovascular disease. Data from the SELECT trial revealed a remarkable 39.1% reduction in hs-CRP levels in the semaglutide group, accompanied by an early separation in cardiovascular event incidence compared to the placebo group. These findings suggest that semaglutide 2.4mg's cardiovascular benefits may extend beyond weight loss and might be partly due to its ability to improve the chronic inflammatory state in obese patients with cardiovascular disease (42).

In recent years, inhibitors targeting NLRP3 inflammatory vesicles have demonstrated potential efficacy in clinical trials. For instance, a small molecule drug (MCC950) that selectively targets NLRP3 inflammatory vesicles can effectively block its activation, thereby significantly delaying the progression of atherosclerosis (43). In streptozotocin-induced diabetic mice with aortic atherosclerotic lesions, MCC950 prevented the formation of atherosclerotic lesions, reduced the expression of inflammatory mediators, and improved vessel wall function (44). In addition, another NLRP3 inflammatory vesicle-targeting drug, quercetin, has been demonstrated to effectively prevent and control neuroinflammatory diseases. Furthermore, quercetin has been shown to significantly reduce plaque area and lipid deposition, stabilize plaques, and inhibit macrophage pyroptosis and NLRP3 expression, ultimately leading to an ameliorative effect on AS in ApoE ^−/−^ mice (45). In addition, selective NLRP3 inhibitors have been studied in phase I-II clinical trials over the past five years. Colchicine, a non-selective NLRP3 inflammatory vesicle inhibitor, has established efficacy in the treatment of pericarditis and is regarded as a standard of care. It is currently approved by the US Food and Drug Administration for reducing the risk of atherosclerotic thrombosis in patients with coronary artery disease (38).

Effective management of inflammation is essential for mitigating the risk of cardiovascular events. The NLRP3 inflammasome, a key player in the inflammatory response linked to cardiovascular disease, presents a promising target for novel therapeutic approaches. Targeted anti-inflammatory treatments against the NLRP3 inflammasome offer significant potential for the development of highly effective CVD therapies.

## 5 Limitations

This study utilized bibliometric methods to analyze research on the NLRP3 inflammasome in cardiovascular diseases retrieved from the Web of Science Core Collection. This analysis aimed to identify the current research landscape, emerging trends, and key areas of focus within this field. However, some limitations should be acknowledged. First, the restriction to a single database (Web of Science) for literature screening stemmed from software limitations that currently prevent simultaneous analysis of multiple sources. While the Web of Science is a well-respected academic database, future studies will explore methods to incorporate additional databases for a more comprehensive analysis. Second, the chosen timeframe (2012–2023) might limit the capture of early keyword usage and the evaluation of long-term trends. Notably, significant advancements have occurred within this field since 2017, and the 12-year window may not fully encompass this progress. Additionally, the relatively small sample size of 516 publications suggests substantial room for future exploration. Finally, the combined use of CiteSpace and Bibliometrix for literature analysis might lead to potential information gaps during the analysis process, introducing some bias into the results. Future work will focus on optimizing the selection of bibliometric methods to address these limitations.

## 6 Conclusions

Bibliometric analysis reveals a promising and rapidly growing field of research investigating the NLRP3 inflammasome in cardiovascular diseases. Notably, the publication volume has reached its peak within the past two years. Examining key contributors, geographic regions, and prominent publications within this field demonstrates a primary focus on coronary heart disease, atherosclerosis, obesity, metabolic syndrome, ASCVD, and related conditions. Recent studies have highlighted the significant roles of NF-κB, oxidative stress, and CRP in the pathogenesis of NLRP3 inflammasome-associated CVDs. A pivotal shift occurred in 2017, with research momentum growing around anti-inflammatory treatment for cardiovascular diseases. This shift coincided with the recognition of hs-CRP as a more reliable biomarker for predicting heart disease risk compared to cholesterol levels. Consequently, the research focus has pivoted towards developing anti-inflammatory therapies in the context of CVD, with particular interest in their synergy with lipid-lowering therapy, as well as their potential to mitigate NLRP3 inflammasome-induced oxidative stress and pyroptosis.
